# Predictive value of the systemic immune–inflammation index for outcomes in large artery occlusion treated with mechanical thrombectomy—a single-center study

**DOI:** 10.3389/fneur.2024.1516577

**Published:** 2025-01-29

**Authors:** Ao Qian, Longyi Zheng, Hui He, Jia Duan, Shuang Tang, Wenli Xing

**Affiliations:** ^1^Department of Cerebrovascular Disease, Suining Central Hospital, Suining, China; ^2^Department of Radiology, School of Medicine, Xiang’an Hospital of Xiamen University, Xiamen University, Xiamen, China; ^3^Department of Outpatient, Suining Central Hospital, Suining, China

**Keywords:** acute ischemic stroke, large artery occlusion, mechanical thrombectomy, systemic immune-inflammation index, mild hypothermia

## Abstract

**Background:**

The systemic immune–inflammation index (SII) is a composite and easily available inflammation index, which can quantitatively reflect the degree of inflammation. This study aims to investigate the predictive value of admission SII for outcomes of large artery occlusion treated with mechanical thrombectomy (MT).

**Methods:**

This retrospective study was conducted at Suining Central Hospital, Sichuan, China. Patients were stratified into quartiles based on their SII. The investigating outcomes included hemorrhagic transformation (HT), malignant brain edema (MBE), 90-day functional outcome, and mortality. The adverse function was defined as the modified Rankin Scale (mRS) score > 2 at the 90-day follow-up. Multivariate analysis was performed to explore the relationships between SII and outcomes. In addition, cases (distinguished from the aforementioned patients) treated with MT + mild hypothermia (MH) were also included to elucidate the relationships between SII/MH and outcomes in a new cohort.

**Results:**

A total of 323 patients treated with MT were included. The observed HT, MBE, adverse function, and mortality rates were 31.9, 25.7, 59.4, and 27.9%, respectively. Multivariate analysis demonstrated that heightened SII was significantly related to HT (odds ratio [OR]: 1.061, 95% confidence interval [CI]: 1.035–1.086, *p* < 0.001), MBE (OR: 1.074, 95% CI: 1.045–1.103, *p* < 0.001), adverse function (OR: 1.061, 95% CI: 1.031–1.092, *p* < 0.001), and mortality (OR: 1.044, 95% CI: 1.018–1.070, *p* = 0.001), after adjusting sex, age, Glasgow Coma Scale (GCS) score at admission, initial National Institutes of Health Stroke Scale (NIHSS) score, baseline Alberta Stroke Program Early Computed Tomography Score (ASPECTS), present HMCAS, occluded vessel region, collateral score and successful revascularization. HT and MBE may partially account for patients with elevated SII’s adverse function and mortality. In addition, with the criterion of baseline ASPECTS ≤ 7, a total of 42 patients treated with MT + MH were enrolled to build up a new cohort combined with 72 patients treated with mere MT. The risk role of SII and protect effect of MH were identified for HT (SII—OR: 1.037, 95% CI: 1.001–1.074; MH—OR: 0.361, 95% CI: 0.136–0.957), MBE (SII—OR: 1.063, 95% CI: 1.019–1.109; MH—OR: 0.231, 95% CI: 0.081–0.653), and mortality (SII—OR: 1.048, 95% CI: 1.011–1.087; MH—OR: 0.343, 95% CI: 0.118–0.994).

**Conclusion:**

Elevated SII was related to HT, MBE, 90-day adverse function, and mortality after MT. The MH may improve prognosis under high inflammation status.

## 1 Introduction

Acute ischemic stroke (AIS) related with large artery occlusion (LAO), is characterized by high morbidity and mortality (1). With the development of interventional technology, mechanical thrombectomy (MT) has become the frontmost therapeutic modality for LAO patients with contraindications or ineffectiveness of thrombolysis (2). Rapid recanalization of occluded arteries and restoring blood flow to salvageable brain tissue can limit the transformation of the ischemic penumbra into infarction (3). However, despite its efficacy, substantial neurological and life-threatening complications still exist. Hemorrhagic transformation (HT) and malignant brain edema (MBE) are common complications after MT that are mainly caused by blood–brain barrier (BBB) disruption (4, 5). Severe HT and MBE prominently increase intracranial pressure, leading to cerebral hernia, and often result in poor functional outcomes that include even death (6). Therefore, identifying predictors of complications and risk factors for poor outcomes may improve patients’ prognoses.

Inflammation plays a pivotal role in the pathophysiology of AIS, involving the progression and resolution of infarction and remodeling and repair of ischemic tissue. However, inflammatory cascade also injuries vascular endothelium and disrupts the integrity of BBB, increasing the risk of HT and MBE (3, 7). Systemic immune–inflammation index (SII) is a composite inflammation index, calculated by combining neutrophil, lymphocyte, and platelet, and quantitatively reflects the inflammatory status (8). Previous studies have found relationships between high levels of SII and poor prognosis in intracerebral hemorrhage (ICH) and subarachnoid hemorrhage (9, 10). Recently, a series of reports have separately put forward that elevated SII is related to HT, MBE, and unfavorable functional outcomes in patients treated with MT (11–13). While none has confirmed these relationships in the same cohort. Therefore, we conducted a retrospective study and set a primary purpose to investigate the predictive value of SII for early complications (HT and MBE) and late outcomes (90-day functional status and mortality). Our secondary objective was to explore the potential mechanism of SII linking to 90-day outcomes.

## 2 Methods

### 2.1 Study population

The institutional ethics committee of our institution approved (No. KYLLK20240133) this study; we retrospectively reviewed the database of the stroke center in Suining Central Hospital, Sichuan, China, and the data of patients undergoing MT for LAO from January 2021 to May 2024 were screened. Patient selection was based on the following inclusion criteria: (1) patients were admitted within 24 h after disease onset; (2) patients with age more than 18 years; and (3) patients suffered from LAO in anterior circulation (internal carotid artery [ICA] or middle cerebral artery [MCA]). The exclusion criteria were as follows: (1) Patients with LAO in the posterior circulation (vertebral artery or basilar artery); (2) patients with apparent infection or immune disease at admission; (3) patients who had comorbidities that required hormone or immunosuppressive agent therapy; (4) patients with progressive or decompensated comorbidities before AIS onset; (5) patients without SII data at admission or outcomes at follow-up; and (6) patients with a history of stroke had residual apparent dysfunction before AIS onset (modified Rankin Scale [mRS] score ≥ 2). In addition, patients with multiple times of MT for re-occlusion of ICA or MCA within the same hospital stay were also excluded, because this may interfere with the predictive value of SII for outcomes.

### 2.2 Patients management

A comprehensive evaluation was deployed immediately after patients were admitted to our emergency, including blood sample collection, consciousness assessment by Glasgow Coma Scale (GCS) score, neurological deficits assessment by the National Institutes of Health Stroke Scale (NIHSS) score, and neuroimaging test. The non-enhanced computed tomography (CT) of the brain was performed to rule out ICH, and CT angiography to confirm the presence of LAO. Intravenous thrombolysis with alteplase was administered within 4.5 h after AIS onset, and MT was carried out for patients with contraindication or futile recanalization of thrombolysis. For LAO with a time window of 4.5–24 h from onset, MT was performed after confirming the mismatch of ischemic and infarction areas evaluated by CT perfusion or MRI/DWI. All MT procedures were performed under general anesthesia using a modern stent retriever and/or direct aspiration technique. Stent implantation or balloon angioplasty was performed for stenosis or dissection at the surgeon’s discretion. The vessel recanalization was measured by modified thrombolysis in cerebral infarction (mTICI) score ≥ 2b was regarded as successful recanalization. Time from onset to recanalization was defined as the time from symptom appearance to successful recanalization or abortion of procedure if successful recanalization was not achieved (14). Planned cranial CT scans were conducted immediately after surgery, 24- and 48-h postprocedure. A CT was also taken whenever there was a change in neurological symptoms.

### 2.3 Data collection

The following clinical and radiological data were collected: demographics, medical history, admission GCS score and NIHSS score, baseline Alberta Stroke Program Early Computed Tomography Score (ASPECTS) on non-enhanced CT before MT, intravenous thrombolysis, laboratory examinations, and procedure details. History of hypertension, diabetes mellitus, and heart disease was determined by self-report, current medication, or prior medical records. The definition of a smoker was smoking at least one cigarette per day for 1 year or more, and the definition of alcoholism was consuming 80 g of liquor per day. The etiology of stroke was assessed using the Trial of OGR 10172 in Acute Stroke Treatment (TOAST) classification (15). Collateral circulation was evaluated based on DSA at the beginning of the MT, categorized as follows: Grade 0: no significant or little filling of the occluded region or less than one-third of the occluded territory; grade 1: collateralization fills less than two-thirds of the occluded territory; and grade 2: collaterals reach more than two-thirds of the occluded territory or the proximal main stem (16). The HT was indicated as the presence of postoperative hemorrhage in the cerebral infarction area. The MBE was defined as follows: (1) More than 50% of middle cerebral artery supply territory had parenchymal hypodensity, accompanied by local brain edema, like lateral ventricle compression and sulcal effacement; and (2) midline shift of >5 mm at septum pellucidum or pineal gland with basal cistern occlusion (17). Two neuroradiologists independently evaluated the perioperative imaging characteristics blind to clinical data, including baseline ASPECTS, hyperdense middle cerebral artery sign (HMCAS), occluded vessel region, collateral score, and postoperative HT and MBE. Any discrepancies were figured out through discussion. The laboratory test data included peripheral blood cell counts, high-sensitivity C-reactive protein (hsCRP), coagulation function, serum electrolyte, liver and kidney function, blood lipid, cholesterol, and glucose. SII was calculated by the following formula: Neutrophil × Platelet/Lymphocyte. Qualified personnel or physicians evaluated the 90-day outcome measures through in-person interviews at the clinic or by telephone. The mRS score was used to assess the 90-day functional outcome, of which mRS score 0–2 was defined as favorable function, and mRS score 3–6 as adverse function (17).

### 2.4 Additional cases treated by mechanical thrombectomy combined with mild hypothermia

From July 2023, a therapeutic method of mild hypothermia (MH) was conducted for patients with LAO treated by MT in our institution. Patients were included if (1) baseline ASPECTS ≤ 7 with or without core infarct volume > 70 mL measured by CT perfusion; and (2) their family members endorsed MH therapy. There was no different preoperative evaluation and surgical procedure strategy for these patients. After the MT procedure, patients were cooled using two cool blankets (Richeng, Changchun, Jilin, China) with a sandwiched method to reach the targeted temperature of 35°C at the posterior pharyngeal wall. All patients were endotracheally intubated with assisted ventilation. Pharmacological sedation with midazolam and analgesia with sufentanil were employed to prevent shivering during hypothermia. Arterial blood gas analysis was performed to monitor PH value, and arterial partial pressure of oxygen and carbon dioxide every 3–6 h. Hematological examinations for peripheral blood cells, hepatorenal function, electrolyte, coagulation function, and myocardial enzyme were scrutinized every 12–24 h. When the target temperature was achieved, the blanket temperature was adjusted to maintain the core temperature at 35.0°C. Slowly rewarming to 36.5°C of core temperature with a controlling rate of no faster than 0.5°C/h was initiated at the final phase of MH therapy by turning off the cooling blankets (18).

### 2.5 Statistical analysis

The patients (without mild hypothermia) were classified according to quartiles of SII levels (Q1–Q4). The categorical variables were presented as numbers (percentage) and were compared using chi-square test or Fisher’s exact test. The continuous variables with normal distribution were expressed as mean ± standard deviation (SD) and compared using analysis of variance (ANOVA) or Student’s *t*-test, otherwise, presented as median with interquartile range, and compared using Kruskal–Wallis test or Mann–Whitney U-test. Univariate analysis was performed for outcomes (HT, MBE, adverse function, and mortality). To avoid overfitting, variables that reached variate significance in all four outcomes were selected to build the “regression model”; in addition, the variable of age was also enrolled. All multivariate analyses in this study were adjusted for this model. The relationships between SII and four outcomes were quantified using multivariate logistic regression, generating odds ratio (OR) and 95% confidence interval (95% CI) after adjusting for multiple covariates. The median SII of each quartile was also treated as a continuous variable for the linear trend test. Weighted linear regression was employed to calculate multicollinearity among variables in the multivariable model, defined as variance inflation factor (VIF) >5. Correlations among variables in the “regression model” were assessed using the Spearman correlation test. Kaplan–Meier curve was depicted to reveal the cumulative incidence of mortality in quartiles of SII using a log–rank test for comparison. The receiver operator characteristic (ROC) curves were plotted to show the diagnostic effect of SII on outcomes. Meanwhile, the AUC values of SII and other composite inflammatory indices that have been verified to correlate with stroke prognosis, including systemic inflammation response index (SIRI; Neutrophil × Monocyte/Lymphocyte), neutrophil-to-lymphocyte ratio (NLR), lymphocyte-to-monocyte ratio (LMR), neutrophil percentage-to-albumin ratio (NPAR), aggregate inflammation systemic index (AISI; Neutrophil × Monocyte × Platelet/Lymphocyte), were calculated and compared using Delong test to explore whether SII possessed better diagnostic effect. Subgroup analysis was performed stratifying sex, age (either below or above 70 years), history of hypertension, TOAST classification (large-artery atherosclerosis [LAA] or others), occluded vessel region (ICA or MCA), collateral score (grades 0 + 1, or 2), and baseline ASPECTS (either below or above 7). The median value was determined as the threshold of SII in the subgroup analysis, using the L50 as a reference. In subgroup analysis, the likelihood ratio tests were employed to probe for interactions between SII and stratified variables. The Sobel test was used for mediation analysis to explore whether HT/MBE mediated elevated SII to 90-day adverse function and mortality.

Since the baseline ASPECTS was the primary indicator for MH, patients underwent mere MT with ASPECTS ≤ 7, and those with MH were selected to form a new cohort. The multivariate logistic regression was performed to explore the role of SII and MH in predicting the four outcomes after controlling for the “regression model.” All statistical analyses were carried out using Statistical Package for the Social Sciences (SPSS) version 25.0 (IBM, Armonk, NY, USA) and R-program version 4.4.1;^1001^ *p* < 0.05 was considered statistically significant.

## 3 Results

### 3.1 Baseline characteristics

**Figure 1:**
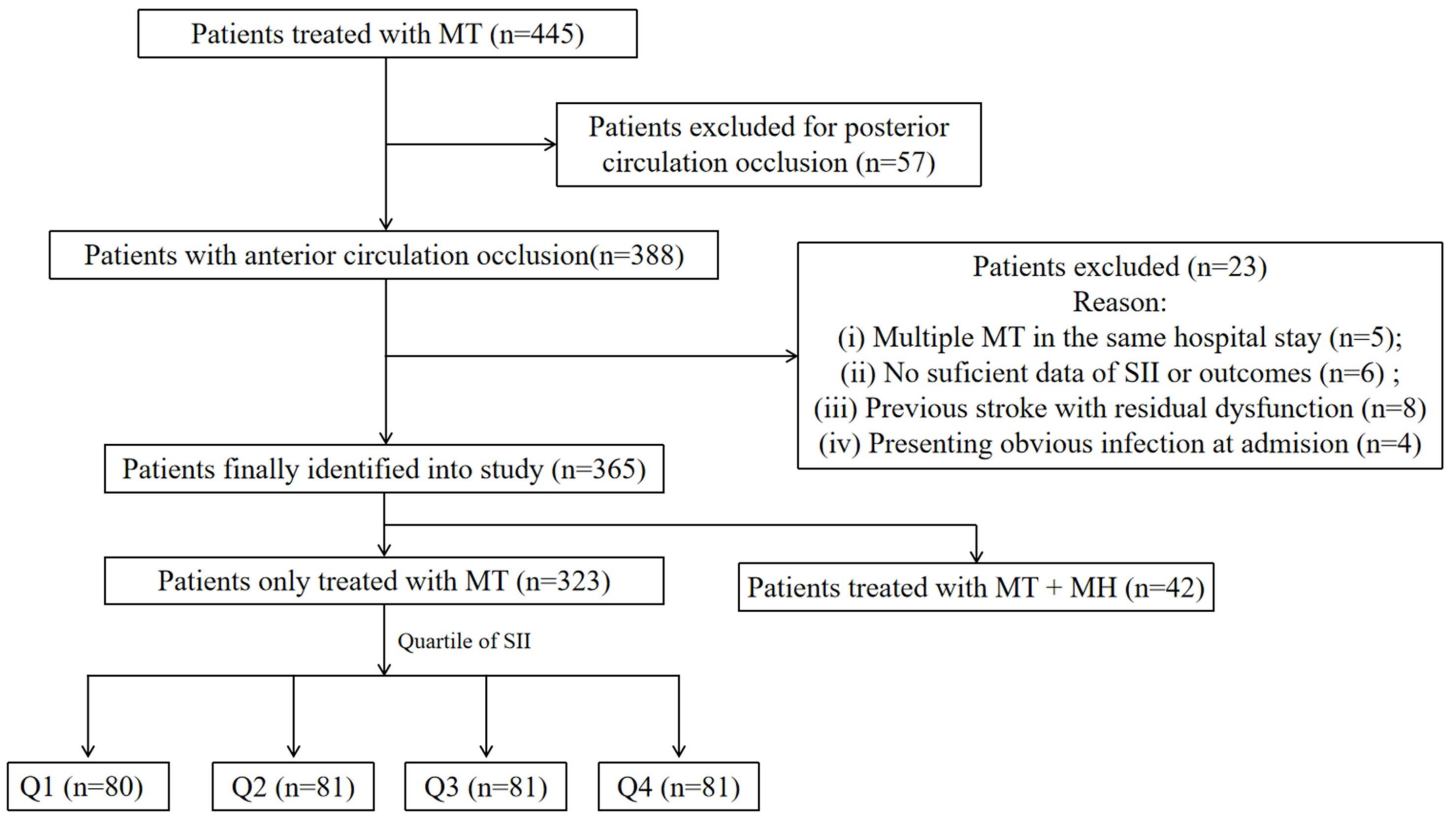
The flow diagram of patient selection.

A total of 445 patients with LAO have been surgically treated in our stroke center, and 323 patients treated with mere MT were eligible for this study. The process of patient selection is depicted in Figure 1. The mean age of patients was 70.73 ± 11.87 years, and 46.7% (151) were women. The ASPECTS, GCS, NIHSS, and NIHSS scores at admission were 8.20 ± 1.27, 12 (IQR: 10–14), and 15 (IQR: 12–19), respectively. One hundred and eighteen (36.5%) patients presented HMCAS. The occluded vessel region was categorized as ICA (124, 38.4%) and MCA (199, 61.6%). The number of patients with collateral scores of grades 0, 1, and 2 was 72 (22.3%), 133 (41.2%), and 118 (36.5%), respectively. A total of 103 (31.9%) patients experienced HT for the early complications and 82 (25.4%) patients suffered from MBE. At 90-day follow-up, 192 (59.4%) patients had adverse function (mRS score: 3–6) and 90 (27.9%) died.

The baseline characteristics of patients classified by quartiles of SII are presented in Table 1. There were 80, 81, 81, and 81 patients in Q1–Q4 Compared to patients with lower SII values, those with higher SII values were more likely to have lower baseline ASEPECTS, longer time from onset to recanalization, and higher serum glucose at admission, while less likely to experience intravenous thrombolysis, and successful revascularization. Critically, these patients possessed a higher proportion of HT, MBE, adverse function, and mortality (all *p* < 0.05).

**Table 1:** Characteristics and outcomes of patients stratified by SII.

Variables	Overall (*n* = 323)	Q1 (*n* = 80)	Q2 (*n* = 81)	Q3 (*n* = 81)	Q4 (*n* = 81)	*p* value
SII	863 (522–1,623)	337 (244–412)	658 (589–750)	1,087 (1,007–1,322)	2,659 (2,124–3,719)	<0.001
**Demongraphics**						
Female, *n* (%)	151 (46.7%)	33 (41.3%)	35 (43.2%)	46 (56.8%)	37 (45.7%)	0.195
Age (years)	70.73 ± 11.87	71.86 ± 12.63	70.80 ± 12.64	70.74 ± 11.01	69.54 ± 11.32	0.682
Smoke, *n* (%)	49 (15.2%)	10 (12.5%)	12 (14.8%)	13 (16.0%)	14 (17.3%)	0.854
Alcoholism, *n* (%)	60 (18.6%)	23 (28.7%)	18 (22.2%)	10 (12.3%)	9 (11.1%)	0.010
**Medical history, *n* (%)**						
Hypertension	156 (48.3%)	35 (43.8%)	43 (53.1%)	38 (46.9%)	40 (49.4%)	0.681
Diabetes mellitus	55 (17.0%)	8 (10.0%)	17 (21.0%)	16 (19.8%)	14 (17.3%)	0.248
Coronary heart disease	40 (12.4%)	8 (10.0%)	11 (13.6%)	10 (12.3%)	11 (13.6%)	0.889
Atrial fibrillation	178 (55.1%)	45 (56.3%)	47 (58.0%)	45 (55.6%)	41 (50.6%)	0.804
Rheumatic heart disease	27 (8.4%)	5 (6.3%)	7 (8.6%)	9 (11.1%)	6 (7.4%)	0.713
Heart failure	18 (5.6%)	5 (6.3%)	5 (6.2%)	5 (6.2%)	3 (3.7%)	0.869
Prior stroke	41 (12.7%)	15 (18.8%)	11 (13.6%)	9 (11.1%)	6 (7.4%)	0.177
Antiplatelet at onset	17 (5.3%)	7 (8.8%)	6 (7.4%)	3 (3.7%)	1 (1.2%)	0.126
Anticoagulant at onset	29 (9%)	13 (16.3%)	7 (8.6%)	3 (3.7%)	6 (7.4%)	0.042
**Clinical and imaging characteristics**						
Systolic pressure at admission (mmhg)	143.50 ± 29.43	146.05 ± 33.53	147.35 ± 28.95	138.00 ± 27.66	142.67 ± 27.08	0.182
Diastolic pressure at admission (mmhg)	83.64 ± 17.60	85.25 ± 22.39	86.63 ± 16.44	79.67 ± 14.42	83.06 ± 15.84	0.065
Intravenous thrombolysis, *n* (%)	100 (31.0%)	26 (32.5%)	34 (42.0%)	24 (29.6%)	16 (19.8%)	0.023
GCS score at admission	12 (10–14)	12 (10–14)	11 (10–13)	12 (9–13)	12 (9–14)	0.381
Initial NIHSS score	15 (12–19)	14 (10–18)	15 (12–18)	16 (14–20)	15 (12–21)	0.036
Baseline ASPECTS	8.20 ± 1.27	8.51 ± 1.14	8.36 ± 1.06	8.04 ± 1.22	7.90 ± 1.55	0.009
Present HMCAS, *n* (%)	118 (36.5%)	27 (33.8%)	30 (37.0%)	27 (33.3%)	34 (42.0%)	0.644
Occluded vessel region, *n* (%)						0.219
ICA	124 (38.4%)	23 (28.7%)	34 (42.0%)	35 (43.2%)	32 (39.5%)	
MCA	199 (61.6%)	57 (71.3%)	47 (58.0%)	46 (56.8%)	49 (60.5%)	
TOAST classification, *n* (%)						0.477
LAA	97 (30.0%)	20 (25%)	25 (30.9%)	22 (27.2%)	30 (37.0%)	
Cardioembolic	199 (61.6%)	55 (68.7%)	50 (61.7%)	49 (60.5%)	45 (55.6%)	
Undetermined or others	27 (8.4%)	5 (6.3%)	6 (7.4%)	10 (12.3%)	6 (7.4%)	
Collateral score, *n* (%)						0.055
Grade 0	72 (22.3%)	12 (15.0%)	12 (14.8%)	22 (27.2%)	26 (32.1%)	
Grade 1	133 (41.2%)	34 (42.5%)	34 (42.0%)	32 (39.5%)	33 (40.7%)	
Grade 2	118 (36.5%)	34 (42.5%)	35 (43.2%)	27 (33.3%)	22 (27.2%)	
**Procedure details**						
Time from onset to recanalization (min)	384.90 ± 127.76	356.54 ± 124.06	376.83 ± 122.49	384.22 ± 123.85	422.79 ± 133.61	0.008
Balloon dilatation, *n* (%)	44 (13.6%)	10 (12.5%)	11 (13.6%)	8 (9.9%)	15 (18.5%)	0.440
Stent implantation, *n* (%)	84 (26.0%)	21 (26.3%)	23 (28.4%)	20 (24.7%)	20 (24.7%)	0.943
Successful revascularization, *n* (%)	294 (91.0%)	77 (96.3%)	75 (92.6%)	74 (91.4%)	68 (84.0%)	0.048
**Laboratory findings**						
Calcium (mmol/L)	2.23 ± 0.17	2.21 ± 0.17	2.23 ± 0.17	2.25 ± 0.17	2.25 ± 0.17	0.450
Sodium (mmol/L)	139.07 ± 3.81	139.52 ± 2.93	139.40 ± 3.69	138.53 ± 4.39	138.86 ± 4.07	0.322
Potassium (mmol/L)	3.83 ± 0.47	3.77 ± 0.46	3.86 ± 0.41	3.82 ± 0.53	3.86 ± 0.48	0.557
Chlorine (mmol/L)	104.34 ± 4.17	105.04 ± 3.66	104.95 ± 3.91	103.31 ± 4.37	104.09 ± 4.51	0.025
hsCRP (mg/L)	5.08 (1.46–13.10)	3.91 (1.18–13.10)	4.35 (1.33–13.10)	5.50 (2.26–13.10)	9.92 (2.05–18.17)	0.179
White blood cell, × 10^9^/L	9.50 ± 3.30	7.57 ± 2.44	8.39 ± 2.34	9.89 ± 2.79	12.13 ± 3.57	<0.001
Neutrophil, × 10^9^/L	7.71 ± 3.36	5.17 ± 2.01	6.57 ± 2.11	8.25 ± 2.65	10.86 ± 3.44	<0.001
Monocyte, × 10^9^/L	0.47 (0.32–0.65)	0.43 (0.30–0.63)	0.47 (0.29–0.59)	0.52 (0.38–0.80)	0.45 (0.27–0.70)	0.016
Lymphocyte, × 10^9^/L	1.34 ± 0.78	1.99 ± 0.88	1.46 ± 0.63	1.13 ± 0.51	0.73 ± 0.30	<0.001
Hemoglobin, × 10^9^/L	125.41 ± 19.24	125.54 ± 17.46	125.74 ± 19.02	124.85 ± 18.62	125.38 ± 21.96	0.991
Platelet, × 10^9^/L	159.40 ± 72.77	119.96 ± 39.25	154.68 ± 76.19	158.32 ± 59.19	204.17 ± 83.04	<0.001
Serum glucose at admission (mmol/L)	8.11 ± 3.11	7.28 ± 2.29	8.48 ± 4.15	8.13 ± 2.46	8.54 ± 3.07	0.032
PT (s)	12.46 ± 1.88	12.35 ± 1.55	12.30 ± 1.95	12.72 ± 2.40	12.48 ± 1.49	0.503
TT (s)	17.3 (16.1–18.2)	17.3 (15.8–18.3)	17.7 (15.9–18.6)	17.1 (16.2–18.1)	17.4 (16.3–18.1)	0.546
APTT (s)	31.64 ± 11.68	30.54 ± 5.34	32.77 ± 20.69	31.15 ± 7.21	32.00 ± 6.20	0.659
Fibrinogen (g/L)	3.04 ± 0.96	2.83 ± 0.80	3.04 ± 0.82	3.05 ± 1.05	3.25 ± 1.10	0.062
INR	1.01 (0.95–1.09)	1.02 (0.96–1.08)	1.00 (0.93–1.08)	1.02 (0.95–1.11)	1.01 (0.96–1.12)	0.696
Albumin (g/L)	39.19 ± 4.32	39.86 ± 3.83	38.83 ± 4.32	39.50 ± 4.21	38.59 ± 4.83	0.218
ALT (U/L)	20 (14–28)	19 (15–25)	20 (13–27)	21 (14–29)	23 (15–31)	0.310
AST (U/L)	27 (21–34)	28 (23–35)	27 (20–32)	25 (21–33)	29 (20–34)	0.365
Serum creatinine (umol/L)	74.96 ± 22.15	73.01 ± 16.34	74.50 ± 18.25	77.08 ± 28.97	74.83 ± 23.00	0.759
Cholesterol (mmol/L)	4.45 ± 1.08	4.28 ± 1.03	4.52 ± 1.07	4.58 ± 1.17	4.42 ± 1.03	0.268
LDLC (mmol/L)	2.75 ± 0.85	2.53 ± 0.77	2.82 ± 0.85	2.87 ± 0.88	2.79 ± 0.86	0.042
HDLC (mmol/L)	1.28 ± 0.35	1.38 ± 0.38	1.25 ± 0.34	1.24 ± 0.31	1.27 ± 0.37	0.032
Triglyceride (mmol/L)	1.25 (0.87–1.97)	1.02 (0.76–1.87)	1.37 (0.90–2.09)	1.36 (0.99–1.81)	1.24 (0.90–1.96)	0.082
**Outcomes, *n* (%)**						
Hemorrhagic transformation	103 (31.9%)	13 (16.3%)	20 (24.7%)	32 (39.5%)	38 (46.9%)	<0.001
Malignant brain edema	83 (25.7%)	6 (7.5%)	17 (21.0%)	24 (28.9%)	36 (44.4%)	<0.001
Adverse outcome, (90-day mRS score 3–6)	192 (59.4%)	30 (37.5%)	43 (53.1%)	57 (70.4%)	62 (76.5%)	<0.001
Mortality at 90-day follow-up	90 (27.9%)	14 (17.5%)	20 (24.7%)	24 (29.6%)	32 (39.5%)	0.016

SII, systemic immune inflammatory index; GCS, Glasgow Coma Scale; NIHSS, National Institute of Health Stroke Scale; ASPECTS, Alberta Stroke Program Early CT Score; HMCAS, hyperdense middle cerebral artery sign; ICA, internal carotid artery; MCA, middle cerebral artery; TOAST, Trial of Org 10,172 in Acute Stroke Treatment; LAA, large-artery atherosclerosis; mTICI, Modified thrombolysis in cerebral infarction; PT, prothrombin time; TT, thrombin time; APTT, activated partial thromboplastin time; INR, international normalized ratio; LDLC, low density lipoprotein cholesterin; HDLC, high density lipoprotein cholesterol; mRS, modified Rankin Scale.

### 3.2 Relationships between SII, HT, MBE, 90-day adverse function, and mortality

**Figure 2:**
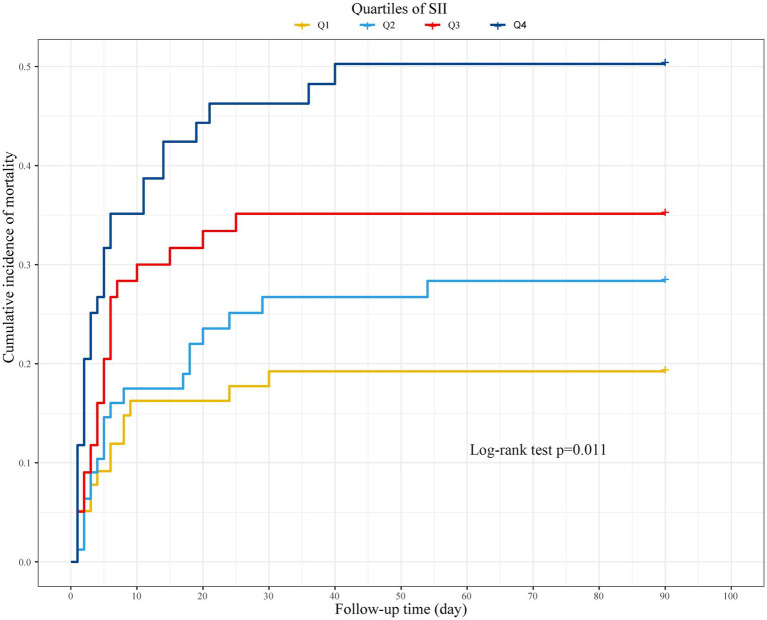
Cumulative incidence of 90-day mortality stratified by quartiles of SII.

The Kaplan–Meier curve demonstrated that individuals with elevated SII exhibited an increased mortality risk at 90-day follow-up (Figure 2). Univariate analysis for HT, MBE, adverse function, and mortality indicated that sex, GCS score at admission, initial NIHSS score, baseline ASPECTS, present HMCAS, occluded vessel region, collateral score, and successful revascularization were the standard significant variables that selected to build up the “regression model” (Supplementary Tables S1–S4), and age was also enrolled. The detailed relationships between SII and outcomes were summarized in Table 2. Multivariate analyses, controlled for sex, age, GCS score at admission, initial NIHSS score, baseline ASPECTS, present HMCAS, occluded vessel region, collateral score, and successful revascularization, demonstrated that patients in the highest quartile had significantly increased risk of HT (OR: 3.691; 95% CI: 1.634–8.340; *p* = 0.002), MBE (OR: 6.755; 95% CI: 2.288–19.940; *p* = 0.001), 90-day adverse function (OR: 4.969; 95% CI: 2.326–10.614; *p* < 0.001), and 90-day mortality (OR: 3.021; 95% CI: 1.159–7.876; *p* = 0.024), compared to those in the lowest quartile, after categorizing the SII as an ordinary variable. When presented as a continuous variable, the SII was also found as a significant predictor of HT (OR: 1.061; 95% CI: 1.035–1.086; *p* < 0.001), MBE (OR: 1.074; 95% CI: 1.045–1.103; *p* < 0.001), 90-day adverse function (OR: 1.061; 95% CI: 1.031–1.092; *p* < 0.001), and 90-day mortality (OR: 1.044; 95% CI: 1.018–1.070; *p* = 0.001). No obvious multicollinearity among variables was detected (VIF from 1.033 to 2.866).

**Table 2:** Crude and adjusted results of SII for outcomes.

Outcomes	Crude			Adjusted*		
	OR (95% CI)	*p* value	P for trend	OR (95% CI)	*p* value	P for trend
**HT**						
Continue variable per 100 unit	1.061 (1.039–1.084)	<0.001		1.061 (1.035–1.086)	<0.001	
Quartile			<0.001			0.001
Q1 (*n* = 80)	Reference			Reference		
Q2 (*n* = 81)	1.690 (0.775–3.685)	0.187		1.419 (0.612–3.290)	0.415	
Q3 (*n* = 81)	3.366 (1.602–7.072)	0.001		2.909 (1.283–6.596)	0.011	
Q4 (*n* = 81)	4.555 (2.179–9.518)	<0.001		3.691 (1.634–8.340)	0.002	
**MBE**						
Continue variable per 100 unit	1.072 (1.048–1.097)	<0.001		1.074 (1.045–1.103)	<0.001	
Quartile			<0.001			<0.001
Q1 (*n* = 80)	Reference			Reference		
Q2 (*n* = 81)	3.276 (1.219–8.808)	0.019		2.796 (0.922–8.476)	0.069	
Q3 (*n* = 81)	4.891 (1.869–12.799)	0.001		3.046 (1.028–9.020)	0.044	
Q4 (*n* = 81)	9.867 (3.853–25.267)	<0.001		6.755 (2.288–19.940)	0.001	
**Adverse outcome**						
Continue variable per 100 unit	1.066 (1.037–1.095)	<0.001		1.061 (1.031–1.092)	<0.001	
Quartile			<0.001			<0.001
Q1 (*n* = 80)	Reference			Reference		
Q2 (*n* = 81)	1.886 (1.006–3.537)	0.048		1.178 (0.860–3.433)	0.126	
Q3 (*n* = 81)	3.958 (2.051–7.640)	<0.001		3.134 (1.516–6.481)	0.002	
Q4 (*n* = 81)	5.439 (2.742–10.787)	<0.001		4.969 (2.326–10.614)	<0.001	
**Mortality**						
Continue variable per 100 unit	1.036 (1.016–1.056)	<0.001		1.044 (1.018–1.070)	0.001	
Quartile			0.002			0.013
Q1 (*n* = 80)	Reference			Reference		
Q2 (*n* = 81)	1.546 (0.718–3.327)	0.266		1.246 (0.492–3.156)	0.642	
Q3 (*n* = 81)	1.985 (0.939–4.195)	0.073		1.564 (0.607–4.032)	0.354	
Q4 (*n* = 81)	3.079 (1.486–6.380)	0.002		3.021 (1.159–7.876)	0.024	

*Adjusted for sex, age, GCS score at admission, initial NIHSS score, baseline ASPECTS, present HMCAS, occluded vessel region, collateral score, and successful revascularization.

SII, systemic immune inflammatory index; HT, hemorrhagic transformation; MBE, malignant brain edema.

**Figure 3:**
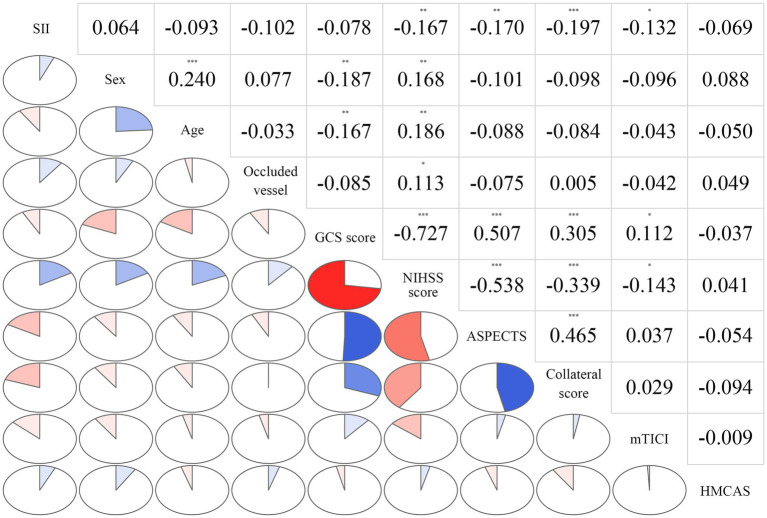
Correlations among variables in the multivariate model. The clockwise and counterclockwise graphs demonstrate positive and negative correlations, respectively. *p < 0.05; **p < 0.01, and ***p < 0.001, respectively.

The correlations among variables in the multivariate model are depicted in Figure 3. The SII was significantly correlated with the initial NIHSS score (*r*_s_ = 0.167, *p* = 0.003), baseline ASPECTS (*r*_s_ = −0.170, *p* = 0.002), collateral score (*r*_s_ = −0.197, *p* < 0.001), and mTICI score (*r*_s_ = −0.201, *p* < 0.001).

### 3.3 SII for predicting the development of HT, MBE, 90-day adverse function, and 90-day mortality

**Figure 4:**
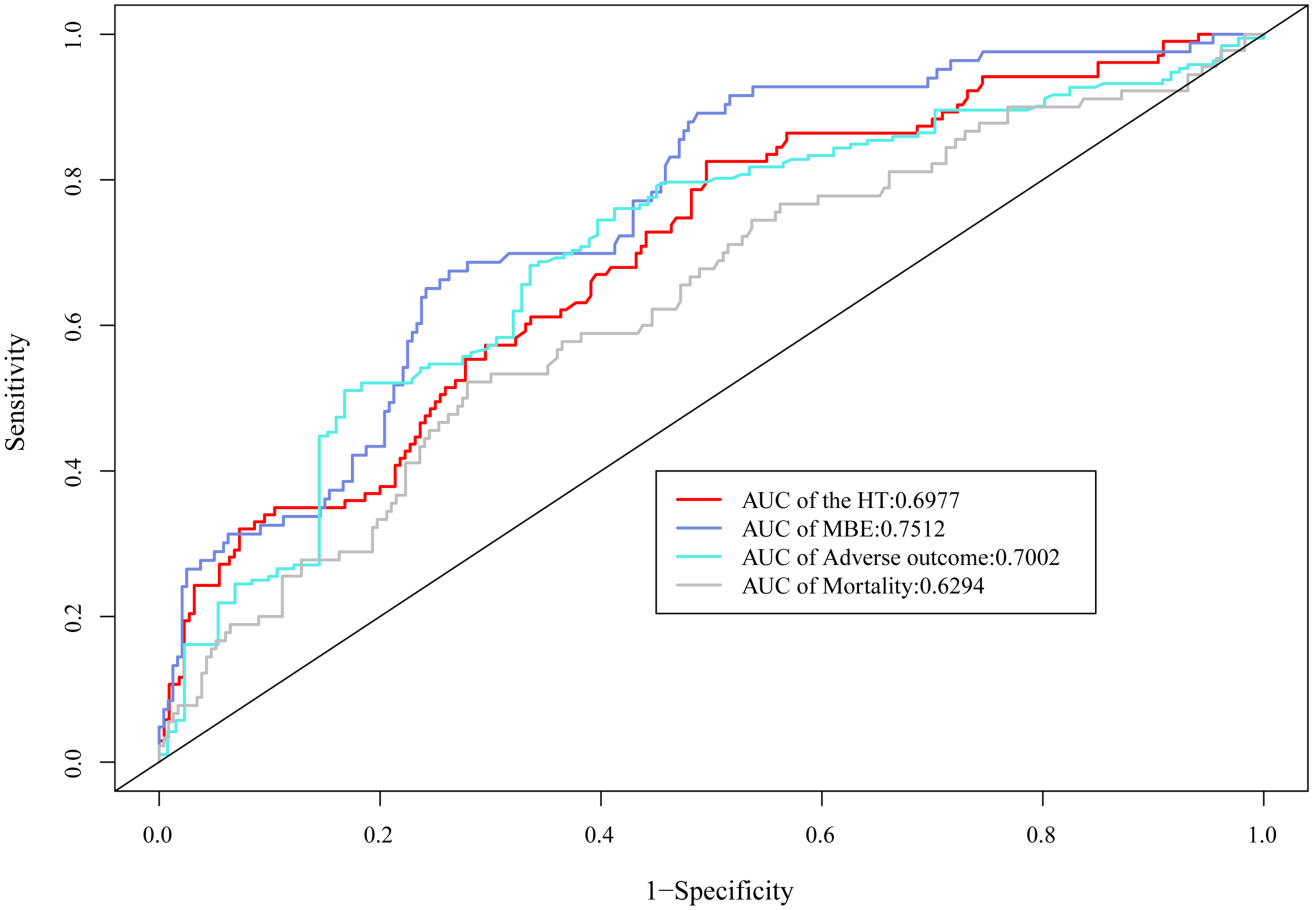
ROC curve demonstrated the moderate diagnostic effect of SII on HT, MBE, 90-day adverse function, and mortality.

The ROC curve suggested the moderate diagnostic performance of SII for HT, MBE, 90-day adverse function, and mortality (AUC 0.6294–0.7512, Figure 4). Importantly, SII showed a better diagnostic effect for these four outcomes than the other inflammatory indices, including SIRI, NLR, LMR, NPAR, and AISI (Table 3).

**Table 3:** Comparisons of diagnostic value for outcomes between SII and other combined inflammatory markers.

	HT		MBE		Adverse outcome		Mortality	
	AUC value	*p* value	AUC value	*p* value	AUC value	*p* value	AUC value	*p* value
SII	0.6977	Reference	0.7512	Reference	0.7002	Reference	0.6294	Reference
SIRI	0.5845	<0.001	0.6093	<0.001	0.6245	0.002	0.5709	0.024
NLR	0.6667	0.078	0.6583	<0.001	0.6697	0.053	0.6012	0.121
LMR	0.5554	<0.001	0.5976	<0.001	0.6221	0.010	0.5940	0.275
NPAR	0.6008	<0.001	0.5525	<0.001	0.6148	0.002	0.5291	<0.001
AISI	0.6157	<0.001	0.6931	0.013	0.6531	0.016	0.5995	0.136

SII, systemic immune inflammatory index; SIRI, systemic inflammation response index; NLR, neutrophil-to-lymphocyte ratio; LMR, lymphocyte-to-monocyte ratio; NPAR, neutrophil percentage-to-albumin ratio; AISI, aggregate inflammation systemic index (neutrophil × monocyte × platelet/lymphocyte); HT, hemorrhagic transformation; MBE, malignant brain edema.

### 3.4 Subgroup analysis

**Figure 5:**
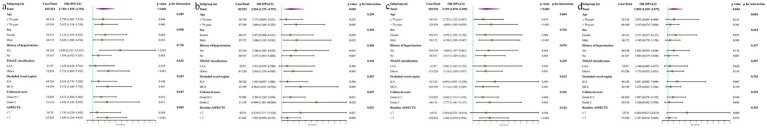
Subgroup analysis evaluated the relationship between SII with HT **(A)**, MEB **(B)**, 90-day adverse function **(C)**, and mortality **(D)** by stratifying age, sex, history of hypertension, TOAST classification, occluded vessel region, collateral score, and baseline ASPECTS, using median value as the threshold of SII.

The subgroup analysis was performed to meticulously evaluate the prognostic utility of SII for predicting the above-mentioned four outcomes, stratifying age, sex, history of hypertension, TOAST classification, occluded vessel region, collateral score, and baseline ASPECTS. No interaction was detected between SII and these variables (all *p* > 0.05). The SII was identified as a substantial predictor of HT, MBE, and 90-day adverse functional outcomes across majority of the patient subgroups. However, no significant correlation was observed between SII and 90-day mortality in all subgroups (all *p* > 0.05, Figure 5).

### 3.5 Mediation analysis of the relationship among SII, HT, MBE, 90-day adverse function, and mortality

**Figure 6:**
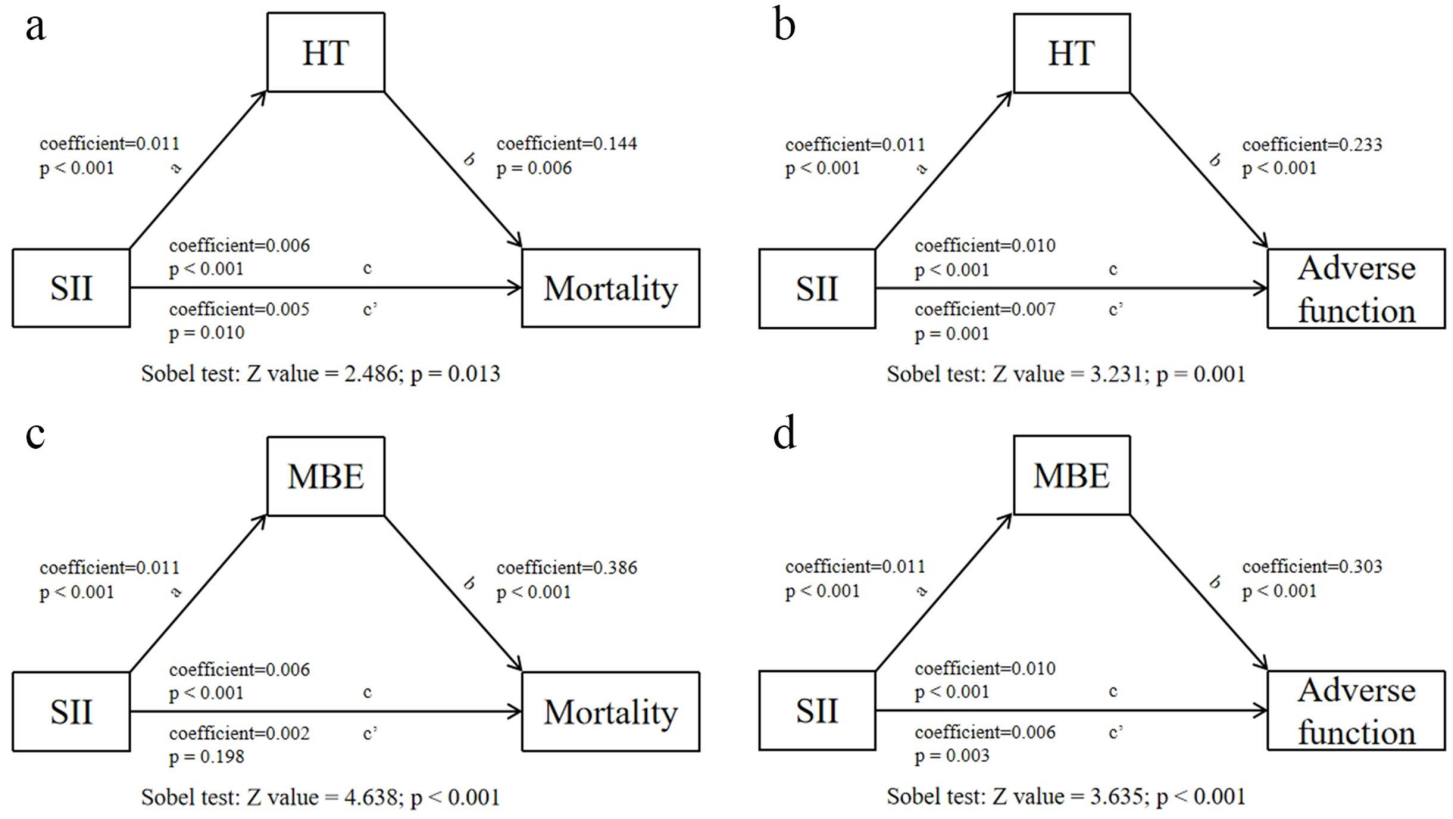
Analysis of the mediating effects of postoperative HT/MBE on the relationship between SII and 90-day adverse function/mortality.

The mediation analysis was conducted to explore whether the relationships between elevated SII and increased risk of 90-day adverse function/mortality were mediated by HT or MBE. Using HT or MBE as a mediator respectively, we revealed the mediating effect of HT and MBE in the effect of the SII on 90-day adverse function and mortality (Figure 6).

### 3.6 Results in the new cohort combined with mild hypothermia

A total of 42 patients were treated with MT + MH, and no severe cardiac arrhythmia, coagulopathy, and hypotension were encountered in these patients. The mean time of inducing hypothermia was 3.4 ± 1.8 h, the duration of maintenance was 53.7 ± 18.1 h, and the period of rewarming was 7.2 ± 2.6 h. Seventy-eight patients who underwent mere MT with ASPECTS ≤ 7 were selected with these 42 patients to form a new cohort. Compared to remaining patients with baseline ASPECTS 8–10, those in the new cohort possessed a significantly high value of SII (759 [IQR: 497–1,467] vs. 1,137 [IQR: 714–2,060], *p* < 0.001). The baseline characteristics of patients treated with or without MH were presented in Supplementary Table S5. A similar value of SII was found in these two groups (MT vs. MT + MH = 1,283 [IQR 706–2,223] vs. 1,066 [IQR 694–1,802], *p* = 0.314). Compared to patients with mere MT, those treated with MT + MH experienced a significantly lower incidence of postoperative HT (48.7% vs. 28.6%, *p* = 0.033), MBE (53.8% vs. 23.8%, 0.002), and 90-day mortality (42.3% vs. 19.0%, *p* = 0.01). In comparison, the rate of 90-day adverse function was similar (82.1% vs. 85.7%, *p* = 0.608). As shown in Table 4, after adjusting for sex, age, GCS score at admission, initial NIHSS score, baseline ASPECTS, present HMCAS, occluded vessel region, collateral score, and successful revascularization, the risk effect of SII and protective effect of MH were observed in HT (SII—OR: 1.037, 95% CI: 1.001–1.074, *p* = 0.047; MH—OR: 0.361, 95% CI: 0.136–0.957, *p* = 0.04), MBE (SII—OR: 1.063, 95% CI: 1.019–1.109, *p* = 0.005; MH—OR: 0.231, 95% CI: 0.081–0.653, *p* = 0.006), and 90-day mortality (SII—OR: 1.048, 95% CI: 1.011–1.087, *p* = 0.011; MH—OR: 0.343, 95% CI: 0.118–0.994, *p* = 0.049). However, the effects were not found in 90-day adverse function (SII—OR: 1.048, 95% CI: 0.983–1.118, *p* = 0.153; MH—OR” 1.214, 95% CI: 0.319–4.622, *p* = 0.776). In addition, other common complications are listed in Table 5. Patients treated with MH + MT developed a significantly higher rate of pneumonia than those treated with only MT (MH + MT vs. MT = 54.8% vs. 30.8%, *p* = 0.010).

**Table 4:** Effects of mild hypothermia and SII on outcomes in patients with baseline ASPECTS ≤ 7.

Outcomes*	Mild hypothermia		SII (Continue variable per 100 unit)	
	OR (95% CI)	*p* value	OR (95% CI)	*p* value
HT	0.361 (0.136–0.957)	0.040	1.037 (1.001–1.074)	0.047
MBE	0.231 (0.081–0.653)	0.006	1.063 (1.019–1.109)	0.005
Adverse outcome	1.214 (0.319–4.622)	0.776	1.048 (0.983–1.118)	0.153
Mortality	0.343 (0.118–0.994)	0.049	1.048 (1.011–1.087)	0.011

*Adjusted for sex, age, GCS at admission, initial NIHSS score, baseline ASPECTS, present HMCAS, occluded vessel region, collateral score and successful revascularization.

SII, systemic immune inflammatory index; HT, hemorrhagic transformation; MBE, malignant brain edema.

**Table 5:** Complications of patients treated with mild hypothermia + mechanical thrombectomy and only mechanical thrombectomy.

	MH + MT (42)	MT (78)	*p* value
Pneumonia	23 (54.8)	24 (30.8)	0.010
Venous thrombosis	5 (11.9)	17 (21.8)	0.182
Urinary tract infection	3 (7.1)	7 (9.0)	1.0
Gastrointestinal bleeding	6 (14.3)	13 (16.7)	0.733
Sepsis	1 (2.4)	1 (1.3)	1.0
Seizure	3 (7.1)	4 (5.1)	0.694

## 4 Discussion

In this study, we investigated the relationship between SII and clinical outcomes in patients with large artery occlusion involving the anterior circulation treated by MT, revealing that elevated SII was linked to both early complications and late prognosis in these patients—even after adjusting for confounding risk factors. Furthermore, SII showed higher diagnostic value than other composite inflammation indices and may lead to 90-day adverse function and mortality by mediating HT and MBE. Therefore, SII may be an independent risk factor for poor prognosis in patients who underwent MT with LAO in the anterior circulation. In addition, first, we put forward that MH could reduce the risk of HT and MEB and be a life-saving measure for patients with high inflammation status.

Increased evidence demonstrates that inflammation plays a crucial role in the occurrence and progression of a series of diseases. As a composite measure derived from neutrophils, lymphocytes, and platelets, SII fully reflects the inflammatory status and has been revealed to be related with the prognosis of a variety of diseases, such as coronary heart disease, non-small cell lung cancer, and liver cirrhosis (19–21). As for stroke, Cao and Song et al. found the relationship between elevated SII and HT in AIS treated with MT, while the relationship between SII and MBE was not explored (11). Ji et al. identified the predictive value of SII for MBE but they did not further investigate the effect of SII on HT (13). Herein, first, we explored the association of SII with HT, MBE, and 90-day outcomes after MT in the same cohort.

Inflammation after AIS occurs early through the activation of both innate and adaptive immune systems. Local hypoxic and proinflammatory milieu stimulates the infiltration of peripheral leukocytes (22). Neutrophils are the earliest leukocyte subtype to react in the blood after AIS. They can rapidly migrate to ischemic brain tissue, aggravating cellular injury and extracellular matrix damage by releasing reactive oxygen species, proteases, and proinflammatory factors (8, 23). Moreover, neutrophils are a vital source of MMP9, contributing to the disruption of blood–brain barrier (BBB) that has been identified as the fundamental mechanism of HT and MBE in patients with AIS (4). In addition, overactivated neutrophils can produce neutrophil extracellular traps (NETs) that trap other blood cells, causing pathological thrombosis and exacerbating neuroinflammatory response (23). Furthermore, the NETs also contribute to the no-reflow phenomenon—defined as inadequate blood flow in certain areas within the brain even after successful recanalization—attributed to local microvascular obstructions, endothelial swelling, or other cellular debris (24, 25). hemostasis, platelets are rich sources of growth factors and play a crucial role in angiogenesis, tissue remodeling, and apoptosis (26). However, platelets are also involved in the inflammation following tissue injury, and platelet activation can directly drive local and systemic inflammatory responses (13, 26). After ischemia/reperfusion, platelets and leukocytes’ interaction aggravates tissue injury through stimulating leukocyte extravasation, oxidative rupture, and microcirculation occlusion (13). In the mice models, despite successful recanalization of the proximal MCA, microvascular occlusion caused by the adhesion of activated platelets and neutrophils was observed in the core infarction and ischemic penumbra area (25, 27). Critically, thrombocytosis and platelet activation are related to elevated disruption of BBB following AIS, which further results in an increased risk of HT and MBE after MT (28). In contrast, lymphocyte is a primary neuroprotective immunoregulator that specific subpopulations can preserve immune homeostasis and relieve cerebral damage by limiting the generation of proinflammatory mediators, regulating the activation of autoreactive cells, and promoting the process of neural repair (3). However, increased levels of catecholamine and cortisol generated by the overactivated sympathetic nervous system and hypothalamic–pituitary–adrenal axis after AIS induce apoptosis and functional inhibition of peripheral lymphocytes (3).

Previous studies on AIS treatment supported that higher inflammatory factors are related with poor prognosis, and are correlated with higher futile recanalization risk when focusing on reperfusion therapy (3, 11–13). However, few of them investigated the mechanism linking early inflammatory status to later outcomes (13). The SII weights the effects of neutrophils, platelets, and lymphocytes for AIS after MT, fully demonstrating the status of systemic inflammation and immune response. Consistent with previous studies, we revealed SII as an independent predictor of HT and MBE after MT, both of which are common complications affecting prognosis (11, 13). Since the value of SII is easily disturbed, it may not be persuasive to explain the effect of SII on later prognosis directly. Therefore, we explored the potential mechanism of SII affecting 90-day functional outcomes and mortality. The mediation analysis confirmed that the effect of an elevated SII on 90-day adverse function and mortality was partially mediated by HT and MBE, which may firstly explain the mechanism that inflammation could affect late prognosis through early complications. Future researches are required to investigate whether early suppression of inflammation reduces the risk of HT and MBE to improve prognosis in patients undergoing MT. However, the results need to be interpreted cautiously because our study only revealed the statistical correlation between SII and outcomes, rather a causal relationship, and further studies elucidating the direct effect are required.

Several immune–inflammation indicators related with stroke prognosis have been proposed in previous reports, such as LMR, NPAR, and AISI (11, 29, 30). The data extracted from the Endovascular Treatment for Acute Basilar Artery Occlusion study demonstrated that a higher neutrophil-to-lymphocyte ratio was related with futile recanalization (31). The systemic inflammatory response index was calculated with the following formula: Neutrophil × Monocyte/lymphocyte, which was related to poor prognosis after MT (3). Compared with complex inflammatory factors calculated by any two indexes, such as NLR, LMR, and NPAR, SII could more accurately evaluate the inflammatory status by assessing neutrophil, lymphocyte, and platelet (32). Considering that inflammatory indicators are susceptible to various factors, such as infection, SII may reduce the bias caused by excessive inclusion of inflammatory indicators, compared with AISI. However, Cao and Song et al. found that NLR had a higher predictive value than SII for symptomatic ICH and 90-day adverse function in patients treated with MT (symptomatic ICH AUC: NLR vs. SII = 0.758 vs. 0.707; 90-day adverse function AUC: NLR vs. SII = 0.662 vs. 0.633) (11). The discrepancy may be generated because only patients who achieved successful recanalization (mTICI of 2b-3) were enrolled in their study. We speculated that patients with futile recanalization had higher thrombus burden, and SII is superior to NLR in reflecting outcomes in these patients (33). In the current study, comparisons of SII with other composite inflammatory markers indicated the higher diagnostic value of SII for outcomes after MT for LAO, further demonstrating the better applicability of SII for clinical guidance.

Mild hypothermia protects brain injury after cerebral ischemia by inhibiting adhesion, aggregation, and infiltration of leukocytes, and blocking the following inflammatory cascade reaction (24). Wang et al. observed that MH significantly reduced neutrophil infiltration and monocyte accumulation after stroke in a model of transient focal cerebral ischemia (34). In mice models, Zhao et al. suggested that MH could reduce the invasion of neutrophils and macrophages to the brain after cerebral ischemia (35). Furthermore, MH could inhibit the aggregation and adhesion of neutrophils formed by no-reflow, which is particularly crucial for highlighting the importance of microvascular patency alongside the opening of large vessels (24). In a rat model of ischemic stroke, Zhao et al. showed that the expression of MMP-9 was significantly decreased in the MH group compared to the control group (36). All the above reports have experimentally confirmed that MH can play a role in brain protection after cerebral ischemia. Multiple prospective studies also identified the relationship between MH clinically and promoted prognosis in patients treated by MT (18, 37, 38). However, the specific indications for MH therapy are still ambiguous, because of the different inclusion criteria in each study. An early pilot study called Cooling for Acute Ischemic Brain Damage (COOL AID) identified the safety and effectiveness of MH therapy for patients treated with intravenous thrombolysis or MT (18). However, Georgiadis et al. reported that MH therapy resulted in higher mortality and higher complication rates compared with hemicraniectomy (39). Different conditions of patients treated with these two methods may be the reason for this discrepancy. In their study, hemicraniectomy was applied for patients with the non-dominant hemisphere affected, while MH therapy was used for patients with the dominant hemisphere affected. In 2016, Schneider et al. also put forward that no benefits of hypothermia in patients treated with hemicraniectomy for large ischemic stroke (40). According to the findings of their study, it might be suggested that later MH (after hemicraniectomy) for patients with large ischemic stroke was even harmful. Among the patients recruited from the Reperfusion and Cooling in Cerebral Acute Ischemia (ReCCLAIM) study, MH was protective against intracerebral hemorrhages after intra-arterial reperfusion therapy (41). Consistent with this study under similar inclusive ASPECTS (ReCCLAIM: 5–7; current study: ≤7), we also observed the protective effect of MH for postoperative HT. As a pilot study in our institution, the MH was only employed for patients with baseline ASPECTS ≤ 7, which has been proven to be an independent predictor of poor outcomes in patients treated with MT (38). In our study, elevated SII was still significantly related to HT, MBE, and 90-day mortality. Furthermore, with a similar value of SII, the MH therapy may have served as a protective factor that significantly decreased the risk of HT, MBE, and 90-day mortality while failing to improve functional outcomes. We speculated that MH suppresses cerebral ischemia’s inflammatory response after MT, causing mitigation of the BBB damage and resulting in reduced risk of HT, MBE, and followed 90-day mortality. However, for patients with large core infarction before MT, extended occlusion has led to irreversible cerebral tissue damage, making ineffectiveness of any subsequent reperfusion efforts, and even if inflammation was inhibited, the functional impairment could not be reversed (24). Nevertheless, MH substantially reduced the risk of HT and MBE, promoted the survival rate of patients, and could also be used as a life-saving measure for patients with high inflammation status.

In mice models, specifically targeting neutrophils with anti-Ly6G could improve penumbral blood flow, and anti-inflammatory treatment could restore microcirculation and decrease no-reflow (42, 43). However, similar therapy has never been evaluated in clinical practice. Although the significant relationship between SII and poor prognosis is observed in the current study, the utility of SII in real-time clinical decision-making remains limited at this stage because only statistical correlation has been identified. Nevertheless, our study may partially reveal the direction of future research, namely, the relationship between anti-inflammation and outcome improvement in patients with MT for LAO. Indeed, this study also had some limitations. First, the single central retrospective design with a limited number of cases in this study relies on the accuracy of data collection and hampers the generalization of these results. Second, the severity and treatment status of comorbidities were failed to be obtained from a significant proportion of patients or their family members. The results may be influenced without considering these variables. Moreover, although there was no obvious collinearity among variables in the multivariate model (all VIF < 5), correlation analysis demonstrated a significant correlation between some variables, which may partially affect the performance of the model’s performance. Third, a single baseline measurement without additional time-point sample collection may not adjust for the variation trend of SII value for predicting outcomes. Potential bias may be generated if the change in SII is also a relevant outcome indicator. Fourth, selection bias in selecting patients for MH may be generated because preoperative ASPECTS based on no-enhanced CT may not be accurate enough to assess the actual ischemia of cerebral tissue. More advanced imaging methods, such as CTP and MRI, and more rigorous inclusion criteria will be implemented for MH therapy. In addition, only patients with ASPECTS ≤ 7 were selected for MH therapy. This hinders us from exploring whether MH therapy could improve prognosis in patients with ASPECTS > 7, and whether these patients in high inflammatory status (high SII) were recommended for MH therapy. Fifth, other potential variables that may be related with outcomes may be ignored to enroll, which may influence the accuracy of ORs of SII predicting outcomes.

## 5 Conclusion

SII is an easily available inflammatory index and can be used as an independent risk factor for HT, MBE, 90-day adverse function, and mortality for patients with LAO in anterior circulation treated by MT, providing a reference for clinical practice. A meticulous protocol is required for patients with high SII value, including close postoperative scrutiny, active therapeutic measures, such as high-dose dehydration or decompressive craniectomy if necessary for MBE, and cautious postoperative anti-platelet/anticoagulation regimens. Postoperative HT and MBE may be the mediators linking SII to 90-day outcomes. With a similar value of SII, MH may improve the prognosis of patients after MT by inhibiting the inflammatory response. However, the current study only provides a preliminary assessment, and more research studies, particularly in randomized controlled trials, are needed to confirm the efficacy and safety of MH in this patient population, and fundamental studies are still required to verify the mechanism.

## Data Availability

The raw data supporting the conclusions of this article will be made available by the authors, without undue reservation.

## Footnotes

^1001^The R-program is an open source software, we have added the a website link of this software: https://www.r-project.org/
