# Supplementary material for: Predictive value of the systemic immune–inflammation index for outcomes in large artery occlusion treated with mechanical thrombectomy—a single-center study
Source: Front Neurol. 2025 Jan 29;15:1516577. doi: 10.3389/fneur.2024.1516577 (PMC11813750; doi:10.3389/fneur.2024.1516577)
Supplement: Supplementary file 1 [file Table_1.DOCX]

Supplementary Table S1. Characteristics between patients with or without hemorrhagic transformation.

| Variables | HT group | non-HT group | P value |
| --- | --- | --- | --- |
| **Patients, n (%)** | 103 (31.9%) | 220 (68.1%) |  |
| **SII** | 1210 (746-2755) | 688 (415-1253) | **< 0.001** |
| **Demongraphics** |  |  |  |
| Female, n (%) | 59 (57.3%) | 92 (41.8%) | **0.009** |
| Age (years) | 71.85 ± 10.53 | 70.20 ± 12.44 | 0.216 |
| Smoke, n (%) | 16 (15.5%) | 33 (15.0%) | 0.901 |
| Alcoholism, n (%) | 16 (15.5%) | 44 (20%) | 0.336 |
| **Medical history, n (%)** |  |  |  |
| Hypertension | 48 (46.6%) | 108 (49.1%) | 0.677 |
| Diabetes mellitus | 21 (20.4%) | 34 (15.5%) | 0.272 |
| Coronary heart disease | 13 (12.6%) | 27 (12.3%) | 0.929 |
| Atrial fibrillation | 59 (57.3%) | 119 (54.1%) | 0.591 |
| Rheumatic heart disease | 12 (11.7%) | 15 (6.8%) | 0.144 |
| Heart failure | 6 (5.8%) | 12 (5.5%) | 0.892 |
| Prior stroke | 11 (10.7%) | 30 (13.6%) | 0.457 |
| Antiplatelet at onset | 5 (4.9%) | 12 (5.5%) | 0.822 |
| Anticoagulant at onset | 7 (6.8%) | 22 (10%) | 0.348 |
| **Clinical and imaging characteristics** |  |  |  |
| Systolic pressure at admission (mmhg) | 145.59 ± 27.53 | 142.52 ± 30.29 | 0.383 |
| Diastolic pressure at admission (mmhg) | 85.50 ± 20.30 | 82.77 ± 16.15 | 0.195 |
| Intravenous thrombolysis, n (%) | 29 (28.2%) | 71 (32.3%) | 0.456 |
| GCS score at admission | 11 (9-13) | 12 (10-14) | **< 0.001** |
| Initial NIHSS score | 17 (13-21) | 14 (10-18) | **< 0.001** |
| Baseline ASPECTS | 7.91 ± 1.44 | 8.33 ± 1.16 | **0.011** |
| Present HMCAS, n (%) | 62 (60.2%) | 56 (25.5%) | **< 0.001** |
| Occluded vessel region, n (%) |  |  | **0.020** |
| ICA | 49 (47.6%) | 75 (34.1%) |  |
| MCA | 54 (52.4%) | 145 (65.9%) |  |
| TOAST classification, n (%) |  |  | 0.985 |
| LAA | 31 (30.1%) | 66 (30%) |  |
| Cardioembolic | 63 (61.2%) | 136 (61.8%) |  |
| Undetermined or others | 9 (8.7%) | 18 (8.2%) |  |
| Collateral score, n (%) |  |  | **0.002** |
| Grade 0 | 35 (34.0%) | 37 (16.8%) |  |
| Grade 1 | 37 (35.9%) | 96 (43.6%) |  |
| Grade 2 | 31 (30.1%) | 87 (39.5%) |  |
| **Procedure details** |  |  |  |
| Time from onset to recanalization (min) | 405.86 ± 119.04 | 375.08 ± 130.75 | **0.043** |
| Balloon dilatation, n (%) | 7 (6.8%) | 37 (16.8%) | **0.014** |
| Stent implantation, n (%) | 21 (20.4%) | 63 (28.6%) | 0.115 |
| Successful Revascularization, n (%) | 87 (84.5%) | 207 (94.1%) | **0.005** |
| **Laboratory findings** |  |  |  |
| Calcium (mmol/L) | 2.24 ± 0.16 | 2.23 ± 0.18 | 0.607 |
| Sodium (mmol/L) | 139.18 ± 3.70 | 139.02 ± 3.87 | 0.735 |
| Potassium (mmol/L) | 3.75 ± 0.43 | 3.86 ± 0.49 | **0.040** |
| Chlorine (mmol/L) | 104.48 ± 3.76 | 104.28 ± 4.35 | 0.689 |
| hsCRP (mg/L) | 9.50 (2.64-13.10) | 3.92 (1.29-13.10) | **0.018** |
| White blood cell, × 10^9^/L | 10.06 ± 3.51 | 9.25 ± 3.18 | **0.039** |
| Neutrophil, × 10^9^/L | 8.55 ± 3.60 | 7.32 ± 3.14 | **0.003** |
| Monocyte, × 10^9^/L | 0.44 (0.28-0.59) | 0.48 (0.33-0.67) | 0.054 |
| Lymphocyte, × 10^9^/L | 1.05 ± 0.57 | 1.47 ± 0.83 | **< 0.001** |
| Hemoglobin, × 10^9^/L | 124.64 ± 17.91 | 125.77 ± 19.86 | 0.624 |
| Platelet, × 10^9^/L | 173.99 ± 76.40 | 152.57 ± 70.15 | **0.013** |
| Serum glucose at admission (mmol/L) | 9.00 ± 3.46 | 7.69 ± 2.85 | **<0.001** |
| PT (s) | 12.42 ± 1.62 | 12.48 ± 2.00 | 0.774 |
| TT (s) | 17.7 (16.5-18.3) | 17.2 (16.0-18.2) | 0.099 |
| APTT (s) | 30.73 ± 6.27 | 32.06 ± 13.48 | 0.338 |
| Fibrinogen (g/L) | 2.92 ± 0.78 | 3.10 ± 1.03 | 0.116 |
| INR | 1.01 (0.94-1.10) | 1.01 (0.96-1.08) | 0.906 |
| Albumin (g/L) | 39.09 ± 4.55 | 39.24 ± 4.23 | 0.776 |
| ALT (U/L) | 23 (15-31) | 19 (14-26) | **0.033** |
| AST (U/L) | 29 (21-36) | 27 (21-33) | 0.518 |
| Serum creatinine (umol/L) | 72.05 ± 21.17 | 76.32 ± 22.52 | 0.106 |
| Cholesterol (mmol/L) | 4.49 ± 1.04 | 4.43 ± 1.10 | 0.620 |
| LDLC (mmol/L) | 2.84 ± 0.84 | 2.71 ± 0.86 | 0.228 |
| HDLC (mmol/L) | 1.23 ± 0.36 | 1.31 ± 0.35 | 0.081 |
| Triglyceride (mmol/L) | 1.34 (0.99-2.02) | 1.19 (0.86-1.89) | 0.056 |

Supplementary Table S2. Characteristics between patients with or without malignant brain edema.

| Variables | MBE group | non-MBE group | P value |
| --- | --- | --- | --- |
| **Patients, n (%)** | 83 (25.7%) | 240 (74.3%) |  |
| **SII** | 1484 (835-3287) | 694 (420-1134) | **< 0.001** |
| **Demongraphics** |  |  |  |
| Female, n (%) | 48 (57.8%) | 103 (42.9%) | **0.019** |
| Age (years) | 69.35 ± 12.05 | 71.20 ± 11.80 | 0.220 |
| Smoke, n (%) | 13 (15.7%) | 36 (15.0%) | 0.885 |
| Alcoholism, n (%) | 10 (12.0%) | 50 (20.8%) | 0.076 |
| **Medical history, n (%)** |  |  |  |
| Hypertension | 43 (51.8%) | 113 (47.1%) | 0.458 |
| Diabetes mellitus | 14 (16.9%) | 41 (17.1%) | 0.964 |
| Coronary heart disease | 7 (8.4%) | 33 (13.8%) | 0.205 |
| Atrial fibrillation | 40 (48.2%) | 138 (57.5%) | 0.142 |
| Rheumatic heart disease | 11 (13.3%) | 16 (6.7%) | 0.062 |
| Heart failure | 3 (3.6%) | 15 (6.3%) | 0.579 |
| Prior stroke | 9 (10.8%) | 32 (13.3%) | 0.557 |
| Antiplatelet at onset | 3 (3.6%) | 14 (5.8%) | 0.575 |
| Anticoagulant at onset | 8 (9.6%) | 21 (8.8%) | 0.807 |
| **Clinical and imaging characteristics** |  |  |  |
| Systolic pressure at admission (mmhg) | 146.49 ± 30.08 | 142.47 ± 29.19 | 0.283 |
| Diastolic pressure at admission (mmhg) | 84.66 ± 15.55 | 83.29 ± 18.27 | 0.540 |
| Intravenous thrombolysis, n (%) | 29 (34.9%) | 71 (29.6%) | 0.363 |
| GCS score at admission | 10 (8-13) | 12 (10-14) | **< 0.001** |
| Initial NIHSS score | 19 (14-23) | 14 (11-17) | **< 0.001** |
| Baseline ASPECTS | 7.51 ± 1.50 | 8.44 ± 1.09 | **< 0.001** |
| Present HMCAS, n (%) | 42 (50.6%) | 76 (31.7%) | **0.002** |
| Occluded vessel region, n (%) |  |  | **< 0.001** |
| ICA | 50 (60.2%) | 74 (30.8%) |  |
| MCA | 33 (39.8%) | 166 (69.2%) |  |
| TOAST classification, n (%) |  |  | **0.020** |
| LAA | 22 (26.5%) | 75 (31.3%) |  |
| Cardioembolic | 48 (57.8%) | 151 (62.9%) |  |
| Undetermined or others | 13 (15.7%) | 14 (5.8%) |  |
| Collateral score, n (%) |  |  | **< 0.001** |
| Grade 0 | 43 (51.8%) | 29 (12.1%) |  |
| Grade 1 | 29 (34.9%) | 104 (43.3%) |  |
| Grade 2 | 11 (13.3%) | 107 (44.6%) |  |
| **Procedure details** |  |  |  |
| Time from onset to recanalization (min) | 393.61 ± 135.41 | 381.88 ± 125.16 | 0.472 |
| Balloon dilatation, n (%) | 6 (7.2%) | 38 (15.8%) | **0.049** |
| Stent implantation, n (%) | 18 (21.7%) | 66 (27.5%) | 0.298 |
| Successful Revascularization, n (%) | 71 (85.5%) | 223 (92.9%) | **0.043** |
| **Laboratory findings** |  |  |  |
| Calcium (mmol/L) | 2.28 ± 0.17 | 2.22 ± 0.17 | **0.003** |
| Sodium (mmol/L) | 139.25 ± 4.37 | 139.01 ± 3.61 | 0.629 |
| Potassium (mmol/L) | 3.90 ± 0.44 | 3.80 ± 0.48 | 0.098 |
| Chlorine (mmol/L) | 104.52 ± 4.72 | 104.28 ± 3.97 | 0.659 |
| hsCRP (mg/L) | 9.50 (2.02-13.10) | 4.15 (1.41-13.10) | 0.170 |
| White blood cell, × 10^9^/L | 9.76 ± 3.45 | 9.41 ± 3.45 | 0.407 |
| Neutrophil, × 10^9^/L | 8.20 ± 3.45 | 7.54 ± 3.29 | 0.125 |
| Monocyte, × 10^9^/L | 0.45 (0.26-0.62) | 0.47 (0.32-0.66) | 0.374 |
| Lymphocyte, × 10^9^/L | 1.07 ± 0.70 | 1.43 ± 0.79 | **< 0.001** |
| Hemoglobin, × 10^9^/L | 125.82 ± 18.86 | 125.27 ± 19.40 | 0.822 |
| Platelet, × 10^9^/L | 202.57 ± 75.98 | 144.48 ± 65.84 | **< 0.001** |
| Serum glucose at admission (mmol/L) | 9.33 ± 4.28 | 7.68 ± 2.46 | **0.001** |
| PT (s) | 12.33 ± 1.39 | 12.51 ± 2.03 | 0.471 |
| TT (s) | 17.2 (16.2-18.1) | 17.4 (16.1-18.2) | 0.710 |
| APTT (s) | 30.44 ± 6.25 | 32.05 ± 13.03 | 0.281 |
| Fibrinogen (g/L) | 2.96 ± 0.87 | 3.07 ± 0.99 | 0.283 |
| INR | 1.00 (0.94-1.09) | 1.01 (0.96-1.09) | 0.439 |
| Albumin (g/L) | 39.65 ± 5.24 | 39.03 ± 3.96 | 0.330 |
| ALT (U/L) | 25 (16-32) | 19 (13-25) | **< 0.001** |
| AST (U/L) | 29 (22-34) | 27 (21-33) | 0.177 |
| Serum creatinine (umol/L) | 73.70 ± 18.50 | 75.39 ± 23.31 | 0.550 |
| Cholesterol (mmol/L) | 4.56 ± 1.08 | 4.41 ± 1.08 | 0.269 |
| LDLC (mmol/L) | 2.91 ± 0.90 | 2.70 ± 0.83 | **0.047** |
| HDLC (mmol/L) | 1.22 ± 0.35 | 1.31 ± 0.35 | 0.051 |
| Triglyceride (mmol/L) | 1.56 (1.09-2.21) | 1.18 (0.86-1.84) | **0.001** |

Supplementary Table S3. Characteristics between patients with adverse or favorable outcome at 90-day follow-up

| Variables | Adverse | Favorable | P value |
| --- | --- | --- | --- |
| **Patients, n (%)** | 192 (59.4%) | 131 (40.6%) |  |
| **SII** | 1106 (681-2133) | 596 (381-1022) | **< 0.001** |
| **Demongraphics** |  |  |  |
| Female, n (%) | 102 (53.1%) | 49 (37.4%) | **0.005** |
| Age (years) | 73.05 ± 11.06 | 67.33 ± 12.24 | **< 0.001** |
| Smoke, n (%) | 31 (16.1%) | 18 (13.7%) | 0.554 |
| Alcoholism, n (%) | 32 (16.7%) | 28 (21.4%) | 0.285 |
| **Medical history, n (%)** |  |  |  |
| Hypertension | 104 (54.2%) | 52 (39.7%) | **0.011** |
| Diabetes mellitus | 38 (19.8%) | 17 (13.0%) | 0.110 |
| Coronary heart disease | 25 (13.0%) | 15 (11.5%) | 0.674 |
| Atrial fibrillation | 102 (53.1%) | 76 (58.0%) | 0.386 |
| Rheumatic heart disease | 16 (8.3%) | 11 (8.4%) | 0.984 |
| Heart failure | 10 (5.2%) | 8 (6.1%) | 0.730 |
| Prior stroke | 28 (14.6%) | 13 (9.9%) | 0.217 |
| Antiplatelet at onset | 10 (5.2%) | 7 (5.3%) | 0.957 |
| Anticoagulant at onset | 20 (10.4%) | 9 (6.9%) | 0.274 |
| **Clinical and imaging characteristics** |  |  |  |
| Systolic pressure at admission (mmhg) | 144.45 ± 29.77 | 142.11 ± 28.97 | 0.485 |
| Diastolic pressure at admission (mmhg) | 84.16 ± 19.12 | 82.89 ± 15.12 | 0.525 |
| Intravenous thrombolysis, n (%) | 56 (29.2%) | 44 (33.6%) | 0.399 |
| GCS score at admission | 11 (9-13) | 13 (11-14) | **< 0.001** |
| Initial NIHSS score | 17 (13-20) | 13 (9-15) | **< 0.001** |
| Baseline ASPECTS | 7.96 ± 1.34 | 8.54 ± 1.08 | **< 0.001** |
| Present HMCAS, n (%) | 79 (41.1%) | 39 (29.8%) | **0.037** |
| Occluded vessel region, n (%) |  |  | **0.030** |
| ICA | 83 (43.2%) | 41 (31.3%) |  |
| MCA | 109 (56.8) | 90 (68.7%) |  |
| TOAST classification, n (%) |  |  | 0.267 |
| LAA | 61 (31.8%) | 36 (27.5%) |  |
| Cardioembolic | 112 (58.3%) | 87 (66.4%) |  |
| Undetermined or others | 19 (9.9%) | 8 (6.1%) |  |
| Collateral score, n (%) |  |  | **0.004** |
| Grade 0 | 54 (28.1%) | 18 (13.7%) |  |
| Grade 1 | 78 (40.6%) | 55 (42.0%) |  |
| Grade 2 | 60 (31.3%) | 58 (44.3%) |  |
| **Procedure details** |  |  |  |
| Time from onset to recanalization (min) | 401.49 ± 128.29 | 360.58 ± 123.47 | **0.005** |
| Balloon dilatation, n (%) | 21 (10.9%) | 23 (17.6%) | 0.089 |
| Stent implantation, n (%) | 51 (26.6%) | 33 (25.2%) | 0.783 |
| Successful Revascularization, n (%) | 166 (86.5%) | 128 (97.7%) | **0.001** |
| **Laboratory findings** |  |  |  |
| Calcium (mmol/L) | 2.27 ± 0.15 | 2.19 ± 0.18 | **< 0.001** |
| Sodium (mmol/L) | 139.02 ± 3.89 | 139.15 ± 3.72 | 0.767 |
| Potassium (mmol/L) | 3.85 ± 0.47 | 3.80 ± 0.48 | 0.322 |
| Chlorine (mmol/L) | 104.38 ± 4.38 | 104.30 ± 3.85 | 0.867 |
| hsCRP (mg/L) | 5.98 (1.97-13.10) | 3.82 (1.27-13.10) | 0.055 |
| White blood cell, × 10^9^/L | 9.72 ± 3.30 | 9.18 ± 3.30 | 0.150 |
| Neutrophil, × 10^9^/L | 8.05 ± 3.34 | 7.21 ± 3.30 | **0.027** |
| Monocyte, × 10^9^/L | 0.45 (0.31-0.66) | 0.48 (0.32-0.63) | 0.880 |
| Lymphocyte, × 10^9^/L | 1.20 ± 0.75 | 1.54 ± 0.79 | **< 0.001** |
| Hemoglobin, × 10^9^/L | 124.17 ± 19.44 | 127.22 ± 18.86 | 0.162 |
| Platelet, × 10^9^/L | 168.25 ± 73.90 | 146.44 ± 69.35 | **0.008** |
| Serum glucose at admission (mmol/L) | 8.65 ± 3.00 | 7.31 ± 3.11 | **< 0.001** |
| PT (s) | 12.46 ± 1.56 | 12.47 ± 2.28 | 0.953 |
| TT (s) | 17.3 (16.2-18.1) | 17.3 (16.1-18.5) | 0.645 |
| APTT (s) | 31.39 ± 9.41 | 32.01 ± 14.41 | 0.639 |
| Fibrinogen (g/L) | 3.05 ± 0.91 | 3.04 ± 1.03 | 0.892 |
| INR | 1.00 (0.95-1.10) | 1.01 (0.97-1.08) | 0.841 |
| Albumin (g/L) | 38.99 ± 4.41 | 39.48 ± 4.19 | 0.329 |
| ALT (U/L) | 21 (14-30) | 18 (14-25) | 0.143 |
| AST (U/L) | 29 (21-35) | 27 (21-31) | 0.322 |
| Serum creatinine (umol/L) | 75.51 ± 23.12 | 74.15 ± 20.72 | 0.589 |
| Cholesterol (mmol/L) | 4.45 ± 0.99 | 4.45 ± 1.20 | 0.954 |
| LDLC (mmol/L) | 2.81 ± 0.81 | 2.66 ± 0.90 | 0.125 |
| HDLC (mmol/L) | 1.22 ± 0.35 | 1.38 ± 0.34 | **< 0.001** |
| Triglyceride (mmol/L) | 1.49 (0.93-2.01) | 1.07 (0.80-1.58) | **< 0.001** |

Supplementary Table S4. Characteristics between patients survival or non-survival at 90-day follow-up

| Variables | Non-survival | Survival | P value |
| --- | --- | --- | --- |
| **Patients, n (%)** | 90 (27.8%) | 233 (72.2) |  |
| **SII** | 1183 (680-2345) | 766 (480-1357) | **< 0.001** |
| **Demongraphics** |  |  |  |
| Female, n (%) | 54 (60.0%) | 97 (41.6%) | **0.003** |
| Age (years) | 75.57 ± 10.28 | 68.86 ± 11.94 | **< 0.001** |
| Smoke, n (%) | 10 (11.1%) | 39 (16.7%) | 0.206 |
| Alcoholism, n (%) | 16 (17.8%) | 44 (18.9%) | 0.819 |
| **Medical history, n (%)** |  |  |  |
| Hypertension | 49 (54.4%) | 107 (45.9%) | 0.169 |
| Diabetes mellitus | 14 (15.6%) | 14 (17.6%) | 0.662 |
| Coronary heart disease | 13 (14.4%) | 27 (11.6%) | 0.485 |
| Atrial fibrillation | 46 (51.1%) | 132 (56.7%) | 0.369 |
| Rheumatic heart disease | 9 (10.0%) | 18 (7.7%) | 0.508 |
| Heart failure | 3 (3.3%) | 15 (6.4%) | 0.276 |
| Prior stroke | 13 (14.4%) | 28 (12.0%) | 0.557 |
| Antiplatelet at onset | 4 (4.4%) | 13 (5.6%) | 0.788 |
| Anticoagulant at onset | 7 (7.8%) | 22 (9.4%) | 0.639 |
| **Clinical and imaging characteristics** |  |  |  |
| Systolic pressure at admission (mmhg) | 150.86 ± 32.03 | 140.66 ± 27.92 | **0.005** |
| Diastolic pressure at admission (mmhg) | 86.19 ± 19.23 | 82.66 ± 16.86 | 0.128 |
| Intravenous thrombolysis, n (%) | 27 (30.0%) | 73 (31.3%) | 0.817 |
| GCS score at admission | 10 (8-12) | 12 (11-14) | **< 0.001** |
| Initial NIHSS score | 18 (14-24) | 14 (11-18) | **< 0.001** |
| Baseline ASPECTS | 7.97 ± 1.48 | 8.29 ± 1.17 | **0.042** |
| Present HMCAS, n (%) | 45 (50.0%) | 73 (31.3%) | **0.002** |
| Occluded vessel region, n (%) |  |  | **0.001** |
| ICA | 48 (53.3%) | 76 (32.6%) |  |
| MCA | 42 (46.7%) | 157 (67.4%) |  |
| TOAST classification, n (%) |  |  | 0.216 |
| LAA | 23 (25.6%) | 74 (31.8%) |  |
| Cardioembolic | 56 (62.2%) | 143 (61.4%) |  |
| Undetermined or others | 11 (12.2%) | 16 (6.8%) |  |
| Collateral score, n (%) |  |  | **0.012** |
| Grade 0 | 29 (32.2%) | 43 (18.5%) |  |
| Grade 1 | 37 (41.1%) | 96 (41.2%) |  |
| Grade 2 | 24 (26.7%) | 94 (40.3%) |  |
| **Procedure details** |  |  |  |
| Time from onset to recanalization (min) | 376.61 ± 100.88 | 388.10 ± 136.80 | 0.410 |
| Balloon dilatation, n (%) | 7 (7.8%) | 37 (15.9%) | 0.057 |
| Stent implantation, n (%) | 17 (18.9%) | 67 (28.8%) | 0.070 |
| Successful Revascularization, n (%) | 70 (77.8%) | 224 (96.1%) | **< 0.001** |
| **Laboratory findings** |  |  |  |
| Calcium (mmol/L) | 2.25 ± 0.15 | 2.23 ± 0.18 | 0.428 |
| Sodium (mmol/L) | 138.87 ± 4.43 | 139.15 ± 3.55 | 0.550 |
| Potassium (mmol/L) | 3.82 ± 0.50 | 3.83 ± 0.46 | 0.915 |
| Chlorine (mmol/L) | 104.44 ± 4.91 | 104.31 ± 3.85 | 0.804 |
| hsCRP (mg/L) | 5.38 (1.97-13.10) | 4.94 (1.43-13.10) | 0.884 |
| White blood cell, × 10^9^/L | 9.48 ± 3.68 | 9.52 ± 3.15 | 0.924 |
| Neutrophil, × 10^9^/L | 7.78 ± 3.66 | 7.68 ± 3.22 | 0.812 |
| Monocyte, × 10^9^/L | 0.42 (0.30-0.60) | 0.48 (0.32-0.67) | 0.291 |
| Lymphocyte, × 10^9^/L | 1.13 ± 0.77 | 1.42 ± 0.77 | **0.003** |
| Hemoglobin, × 10^9^/L | 123.57 ± 17.93 | 126.12 ± 19.71 | 0.286 |
| Platelet, × 10^9^/L | 174.03 ± 76.37 | 153.75 ± 70.69 | **0.024** |
| Serum glucose at admission (mmol/L) | 9.07 ± 3.36 | 7.73 ± 2.93 | **< 0.001** |
| PT (s) | 12.22 ± 1.64 | 12.56 ± 1.96 | 0.145 |
| TT (s) | 17.3 (15.9-18.1) | 17.3 (16.2-18.3) | 0.537 |
| APTT (s) | 29.94 ± 6.41 | 32.29 ± 13.12 | 0.105 |
| Fibrinogen (g/L) | 2.98 ± 0.81 | 3.07 ± 1.01 | 0.438 |
| INR | 1.01 (0.95-1.07) | 1.01 (0.96-1.10) | 0.277 |
| Albumin (g/L) | 39.18 ± 4.58 | 39.19 ± 4.23 | 0.984 |
| ALT (U/L) | 25 (15-31) | 19 (14-26) | **0.042** |
| AST (U/L) | 30 (23-36) | 26 (20-32) | **0.005** |
| Serum creatinine (umol/L) | 77.23 ± 23.85 | 74.08 ± 21.46 | 0.252 |
| Cholesterol (mmol/L) | 4.47 ± 0.92 | 4.44 ± 1.14 | 0.802 |
| LDLC (mmol/L) | 2.85 ± 0.80 | 2.71 ± 0.87 | 0.207 |
| HDLC (mmol/L) | 1.25 ± 0.36 | 1.30 ± 0.35 | 0.255 |
| Triglyceride (mmol/L) | 1.44 (0.92-2.01) | 1.18 (0.86-1.91) | **0.043** |

Supplementary Table S5. Comparison between mild hypothermia group and mere mechanical thrombectomy group with patients having baseline ASPECTS ≤ 7.

| Variables | Mild hypothermia | General method | P value |
| --- | --- | --- | --- |
| **Patients, n (%)** | 42 (35%) | 78 (65%) |  |
| **SII** | 1066 (694-1802) | 1283 (706-2223) | 0.314 |
| **Demongraphics** |  |  |  |
| Female, n (%) | 26 (61.9%) | 41 (52.6%) | 0.326 |
| Age (years) | 73.31 ± 7.43 | 73.45 ± 10.63 | 0.940 |
| Smoke, n (%) | 8 (19.0%) | 12 (15.4%) | 0.608 |
| Alcoholism, n (%) | 8 (19.0%) | 7 (9.0%) | 0.112 |
| **Medical history, n (%)** |  |  |  |
| Hypertension | 24 (57.1%) | 35 (44.9%) | 0.200 |
| Diabetes mellitus | 7 (16.7%) | 14 (17.9%) | 0.860 |
| Coronary heart disease | 4 (9.5%) | 9 (11.5%) | 0.735 |
| Atrial fibrillation | 23 (54.8%) | 36 (46.2%) | 0.368 |
| Rheumatic heart disease | 0 (0%) | 8 (10.3%) | 0.049 |
| Heart failure | 3 (7.1%) | 3 (3.8%) | 0.421 |
| Prior stroke | 7 (16.7%) | 10 (12.8%) | 0.564 |
| Antiplatelet at onset | 2 (4.8%) | 2 (2.6%) | 0.611 |
| Anticoagulant at onset | 2 (4.8%) | 4 (5.1%) | 1.0 |
| **Clinical and imaging characteristics** |  |  |  |
| Systolic pressure at admission (mmhg) | 151.33 ± 27.38 | 140.01 ± 26.71 | 0.030 |
| Diastolic pressure at admission (mmhg) | 88.33 ± 15.74 | 81.27 ± 13.62 | 0.012 |
| Intravenous thrombolysis, n (%) | 15 (35.7%) | 22 (28.2%) | 0.396 |
| GCS score at admission | 9 (7-10) | 9 (7-11) | 0.813 |
| Initial NIHSS score | 21 (17-24) | 22 (18-28) | 0.285 |
| Baseline ASPECTS | 6.31 ± 0.92 | 6.44 ± 0.75 | 0.419 |
| Present HMCAS, n (%) | 17 (40.5%) | 31 (39.7%) | 0.938 |
| Occluded vessel region, n (%) |  |  | 0.319 |
| ICA | 25 (59.5%) | 39 (50%) |  |
| MCA | 17 (40.5%) | 39 (50%) |  |
| TOAST classification, n (%) |  |  | 0.708 |
| LAA | 16 (38.7%) | 24 (30.8%) |  |
| Cardioembolic | 23 (54.8%) | 47 (60.3%) |  |
| Undetermined or others | 3 (7.1%) | 7 (9.0%) |  |
| Collateral score, n (%) |  |  | 0.330 |
| Grade 0 | 26 (61.9%) | 56 (71.8%) |  |
| Grade 1 | 10 (23.8%) | 17 (21.8%) |  |
| Grade 2 | 6 (14.3%) | 5 (6.4%) |  |
| **Procedure details** |  |  |  |
| Time from onset to recanalization (min) | 438.98 ± 200.62 | 389.97 ± 117.18 | 0.151 |
| Balloon dilatation, n (%) | 10 (23.8%) | 4 (5.1%) | 0.005 |
| Stent implantation, n (%) | 10 (23.8%) | 14 (17.9%) | 0.444 |
| Successful Revascularization, n (%) | 39 (92.9%) | 67 (85.9%) | 0.374 |
| **Laboratory findings** |  |  |  |
| Calcium (mmol/L) | 2.23 ± 0.11 | 2.25 ± 0.17 | 0.347 |
| Sodium (mmol/L) | 138.39 ± 3.22 | 139.71 ± 4.80 | 0.113 |
| Potassium (mmol/L) | 3.79 ± 0.43 | 3.83 ± 0.50 | 0.694 |
| Chlorine (mmol/L) | 104.57 ± 3.83 | 104.62 ± 4.89 | 0.958 |
| hsCRP (mg/L) | 8.36 (4.60-11.67) | 10.27 (2.14-14.27) | 0.775 |
| While blood cell, × 10^9^/L | 9.47 ± 3.34 | 9.75 ± 3.38 | 0.664 |
| Neutrophil, × 10^9^/L | 7.75 ± 3.14 | 8.13 ± 3.57 | 0.555 |
| Monocyte, × 10^9^/L | 0.42 (0.27-0.54) | 0.47 (0.32-0.64) | 0.122 |
| Lymphocyte, × 10^9^/L | 1.23 ± 0.56 | 1.12 ± 0.72 | 0.372 |
| Hemoglobin, × 10^9^/L | 124.29 ± 12.28 | 123.62 ± 17.92 | 0.810 |
| Platelet, × 10^9^/L | 176.45 ± 61.89 | 162.90 ± 66.57 | 0.278 |
| Serum glucose at admission (mmol/L) | 8.82 ± 3.57 | 8.53 ± 3.33 | 0.656 |
| PT (s) | 11.54 ± 0.83 | 12.54 ± 1.34 | < 0.001 |
| TT (s) | 16.9 (15.9-18.2) | 17.4 (16.3-18.1) | 0.344 |
| APTT (s) | 26.82 ± 2.98 | 31.67 ± 6.25 | < 0.001 |
| Fibrinogen (g/L) | 3.14 ± 1.14 | 3.05 ± 0.81 | 0.608 |
| INR | 1.02 (0.95-1.06) | 1.00 (0.96-1.08) | 0.772 |
| Albumin (g/L) | 37.85 ± 5.81 | 39.21 ± 4.48 | 0.192 |
| ALT (U/L) | 15 (12-31) | 23 (16-29) | 0.054 |
| AST (U/L) | 26 (20-34) | 30 (23-37) | 0.151 |
| Serum creatinine (umol/L) | 64.74 ± 25.21 | 77.54 ± 19.89 | 0.003 |
| Cholesterol (mmol/L) | 4.65 ± 1.02 | 4.46 ± 1.06 | 0.339 |
| LDLC (mmol/L) | 2.96 ± 0.72 | 2.67 ± 0.84 | 0.054 |
| HDLC (mmol/L) | 1.40 ± 0.31 | 1.29 ± 0.35 | 0.090 |
| Triglyceride (mmol/L) | 1.28 (0.93-1.57) | 1.28 (0.90-2.12) | 0.594 |
| Outcomes |  |  |  |
| HT | 12 (28.6%) | 38 (48.7%) | 0.033 |
| MBE | 10 (23.8%) | 42 (53.8%) | 0.002 |
| Adverse outcome | 36 (85.7%) | 64 (82.1%) | 0.608 |
| Mortality | 8 (19.0%) | 33 (42.3%) | 0.010 |
